# Supplementary figures and images for: Kit Ligand and Kit receptor tyrosine kinase sustain synaptic inhibition of Purkinje cells
Source: eLife. 2024 Mar 27;12:RP89792. doi: 10.7554/eLife.89792 (PMC10972566; doi:10.7554/eLife.89792)

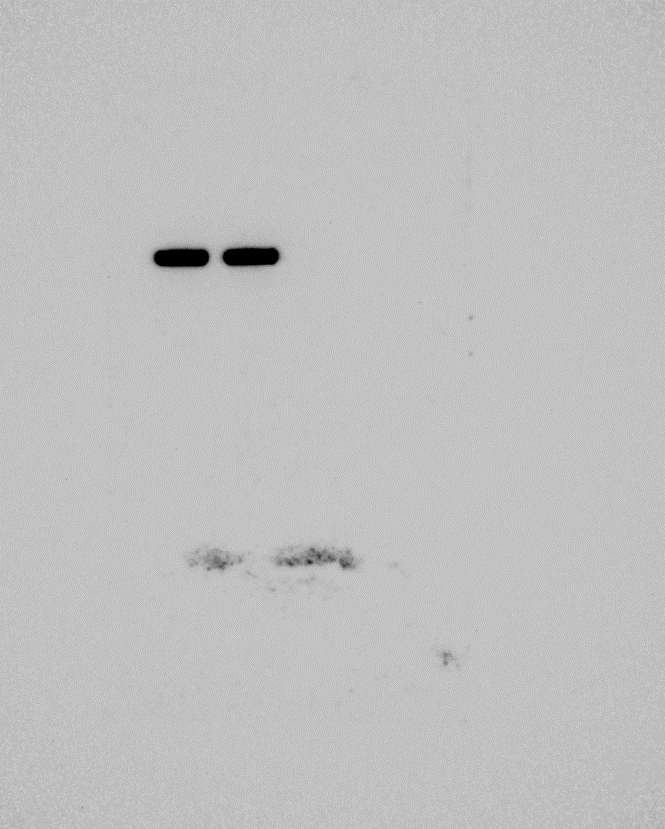

Supplement: Figure 1—source data 1. [file elife-89792-fig1-data1.zip › Figure 1 source data 1.tif]

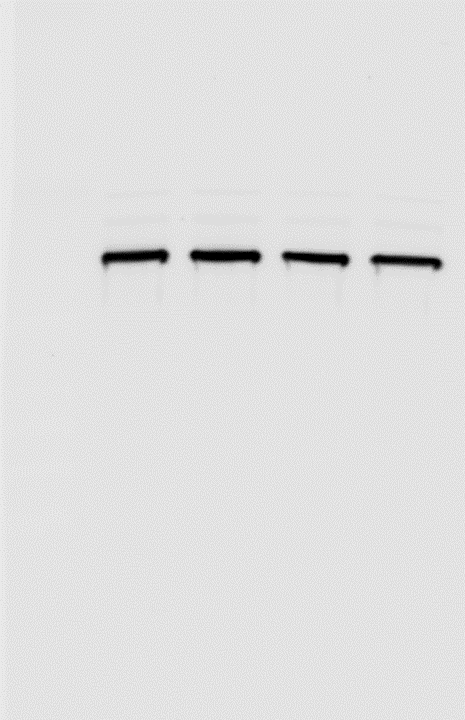

Supplement: Figure 1—source data 2. [file elife-89792-fig1-data2.zip › Figure 1 source data 2.tif]

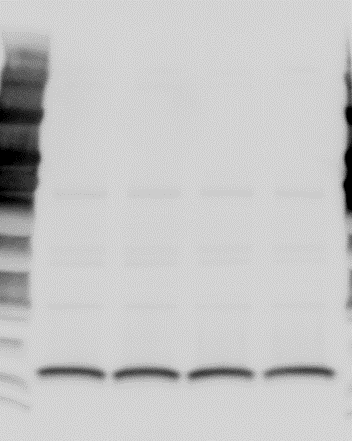

Supplement: Figure 1—source data 3. [file elife-89792-fig1-data3.zip › Figure 1 source data 3.tif]

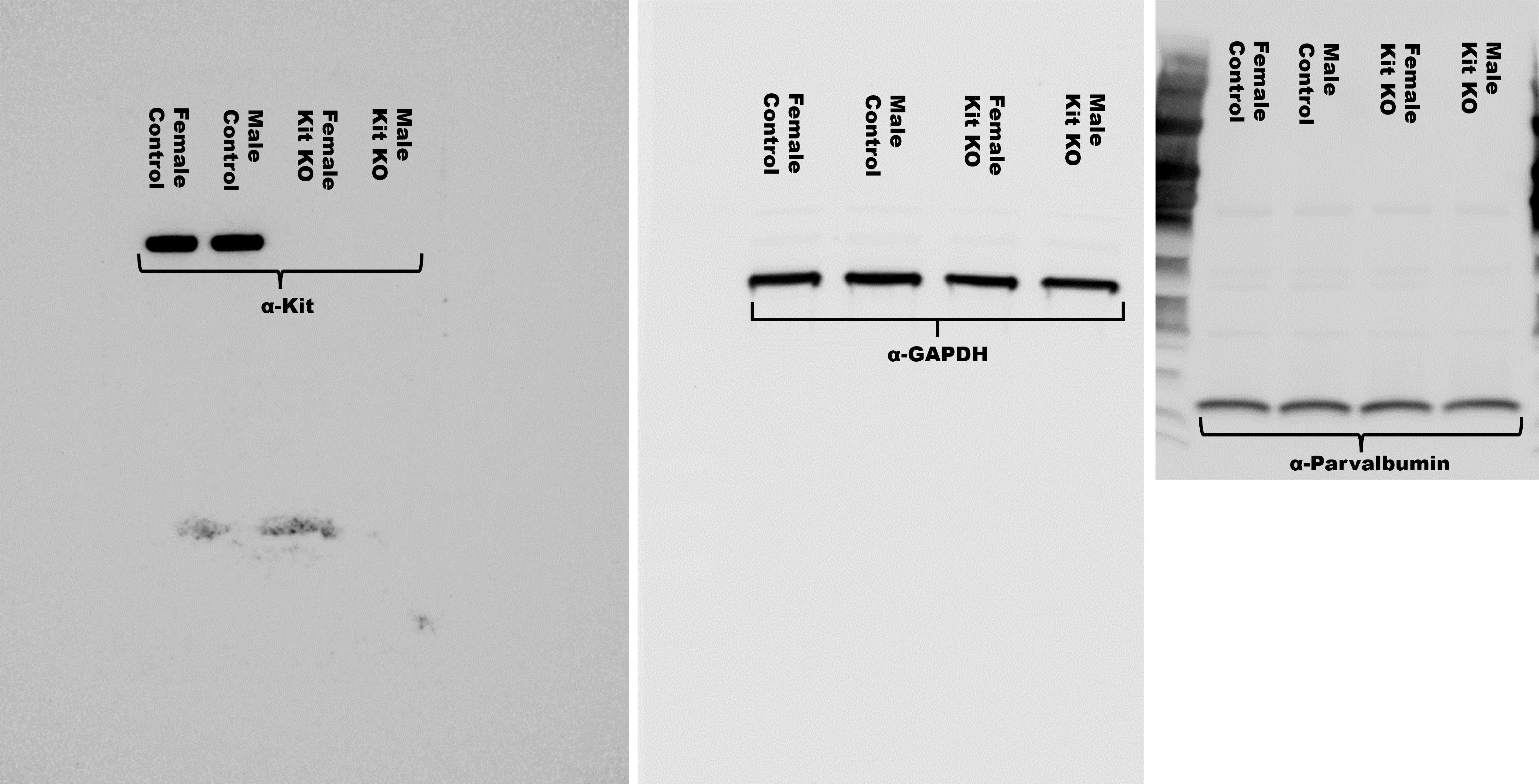

Supplement: Figure 1—source data 4. [file elife-89792-fig1-data4.zip › Figure 1 Source Data 4 Labeled Blots.tif]
